# Anti-Inflammatory, Antipyretic, and Analgesic Potential of Chitin and Chitosan Derived from Cockroaches (*Periplaneta americana*) and Termites

**DOI:** 10.3390/jfb15030080

**Published:** 2024-03-21

**Authors:** Khushbakht Asad, Sumaira Shams, Eliana Ibáñez-Arancibia, Patricio R. De los Ríos-Escalante, Farhad Badshah, Farooq Ahmad, Muhammad Salman Khan, Asar Khan

**Affiliations:** 1 Department of Zoology, Abdul Wali Khan University Mardan, Mardan 23200, Pakistan; khushbakhtasad7@gmail.com (K.A.); farhad.badshah@awkum.edu.pk (F.B.); farooqahmad@awkum.edu.pk (F.A.); salman.khan@awkum.edu.pk (M.S.K.); asar1056@gmail.com (A.K.); 2 PhD Program in Sciences Mentioning Applied Molecular and Cell Biology, La Frontera University, Temuco 4780000, Chile; e.ibanez05@ufromail.cl; 3 Laboratory of Engineering, Biotechnology and Applied Biochemistry—LIBBA, Department of Chemical Engineering, Faculty of Engineering and Science, La Frontera University, Temuco 4780000, Chile; 4 Department of Biological and Chemical Sciences, Faculty of Natural Resources, Catholic University of Temuco, Temuco 4780000, Chile; prios@uct.cl; 5 Nucleus of Environmental Sciences, Faculty of Natural Resources, Catholic University of Temuco, Temuco 4780000, Chile; 6 State Key Laboratory of Animal Biotech Breeding, Institute of Animal Science, Chinese Academy of Agricultural Sciences, Beijing 100193, China

**Keywords:** *Periplaneta americana*, biopolymers, chitosan, chitin, antipyretic, demineralization, deproteinization

## Abstract

The chitin and chitosan biopolymers are extremely valuable because of their numerous industrial and pharmacological uses. Chitin and chitosan were extracted from the exoskeleton of *Periplaneta americana* (cockroaches) and termites using various acid and alkali techniques. The extraction process involves an initial demineralization step, during which integument dry powder was subjected to 500 mL (2.07 mol/L) of concentrated HCl at 100 degrees Celsius for 30 min, followed by meticulous rinsing with distilled water to restore the pH to its baseline. Deproteinization was conducted at 80 degrees Celsius using 500 mL (1 mol/L) of NaOH solution, which was repeated for 24 h. A total of 250 mL (0.06 mol/L) of NaOH was added at 100 degrees Celsius for 4 h to obtain chitosan, followed by extensive washing and subsequent drying. FTIR analysis was used to identify the functional groups in *Periplaneta americana* and termites. The crystallinity of these biopolymers, which have a face-centered cubic structure, was determined by X-ray diffraction analysis. This study assessed the analgesic properties of chitin and chitosan via an acetic-acid-induced writhing test in mice, revealing a significant reduction in writhing behavior following the chitin and chitosan extract. Notably, chitin exhibits the highest degree of analgesic activity compared to chitosan. Both chitin and chitosan show anti-inflammatory effects, with chitosan absorbing proton ions at sites of inflammation, while chitin effectively inhibits ear edema and elicits an analgesic response in mice. Furthermore, the present study revealed antipyretic activity, with termite chitin demonstrating the most significant effect at a concentration of 500 µL/mL, followed by chitosan and chitin at 100 µL/mL. These findings indicate the potential of using chitin and chitosan derived from termites and *Periplaneta americana* as natural anti-inflammatory compounds, implying prospective uses in anti-inflammatory, antipyretic, and analgesic capabilities.

## 1. Introduction

Chitin represents one of the most widespread organic compounds on Earth, after cellulose. Biopolymers and bio-based polymers are gaining attention in a renewed focus on environmentally-conscious technology due to their practicality and improved biodegradability over synthetic polymers. Bio-based polymers are gaining prominence due to their distinctive pharmacological and physical attributes [1,2]. In broad terms, chitin is found in the bodies of certain mushrooms, yeasts, crustaceans, and green algae, as well as in insect cuticles [1,3]. Chitosan ranks as the second most prevalent polysaccharide obtained from the exoskeleton of arthropods [4]. The bodies of organisms typically comprise polysaccharides with a mushy appearance. Notably, chitosan is used within the exoskeletons of insects and crustaceans [5]. Chitosan, a hydrophilic substance, is obtained from chitin polysaccharide via deacetylation. Considering the charge density on the cell surface influences chitosan’s adsorption, strengthening it enhances the ability to permeate the bacterial membrane, modifying its structure simultaneously [6]. Chitin polysaccharide functions like cellulose even though it has specific distinctive characteristics.

Given that chitin is hydrophobic, its potential to dissolve in organic solvents and water is constrained. When dispersed in water, it can be found in mineral acid solutions, hexafluoropropanol, hexafluoroacetone, chloroalcohols, and dimethylacetamide with 5% lithium chloride [7]. Chitosan disintegrates in de-acetylated chitin derivatives, such as acetic and formic acids. Recently, it was discovered that chitosan is used in formulations for controlled drug release and that it can gel in N-methylmorpholine N-oxide. Chitosan molecules are recognized to exhibit fungicidal and bactericidal characteristics [8]. A lengthy deacetylation process is required for chitin to produce chitosan. The protein undergoes deacetylation at a temperature of 100 degrees Celsius using a sodium hydroxide solution. The degree of deacetylation (DDA) content in chitosan synthesis exhibits variability, which is influenced to different extents by temperature and the concentration of sodium hydroxide. The solubility of chitosan in aqueous or acidic solutions is contingent upon the concentration of DDA [9]. Organic solvents do not dissolve chitin, although inorganic acids such as hydrochloric, sulfuric, or phosphoric acid can dissolve chitin. Despite being dangerous, poisonous, and corrosive, these solvents can effectively dissolve chitin [10,11]. Various factors, such as pH solution, temperature, ionic strength, molecular weight, and chitosan and chitin concentration, have been addressed in the literature. The solubility of chitin is determined by the degree of acetylation; specifically, the polymer DE acetylation, as shown by the presence of N-acetyl amino groups [12]. Chitosan and chitin are separated through demineralization, deproteinization, and deacetylation processes, synthesized by termites and *Periplaneta americana* using solvents like acetone, hydrogen peroxide, and sodium hypochlorite [1]. *Periplaneta americana* have flourished globally, particularly in the tropics and subtropics, and their habitats vary considerably [13]. In addition to residential locations, *Periplaneta americana* was a worldwide pest found in facilities that house food, bakeries, restaurants, basements, and sewers [14]. Termites are essential for ecosystem maintenance and colonization globally, except in Antarctica. They range from small colonies to large societies and undergo incomplete metamorphosis like ants [15]. This study aims to identify biological sources with potentially useful pharmacological characteristics. The current study aimed to examine the possible anti-inflammatory, analgesic, and antipyretic effects of termite and *Periplaneta americana* extracts on mice. Typically, the biological sources were low-cost, health-promoting drugs with no side effects.

## 2. Methods and Materials

### 2.1. Collection of Isoptera (Termite) and Periplaneta americana

The study and animal-related experiments were conducted following the Declaration of Helsinki, and the protocols were approved by the Ethics Committee of the Department of Zoology, Abdul Wali Khan University Mardan (Approval No. AWZ20231102). The insect species *Periplaneta americana* were collected from the Mardan district. The *Periplaneta americana* specimens were primarily obtained from the Khyber Teaching Hospital (KTH) in Peshawar and homes (kitchen), and termites were collected from woods in the Gujrat area of district Mardan. The specimens were then identified by the pharmacy department of Abdul Wali Khan University Mardan. Thirty-six mice (all female and about 4–5 weeks old) were used in this study, and mice were bought from the Peshawar culturing laboratory.

### 2.2. Preparation of Insect Extracts

**Figure 1:**
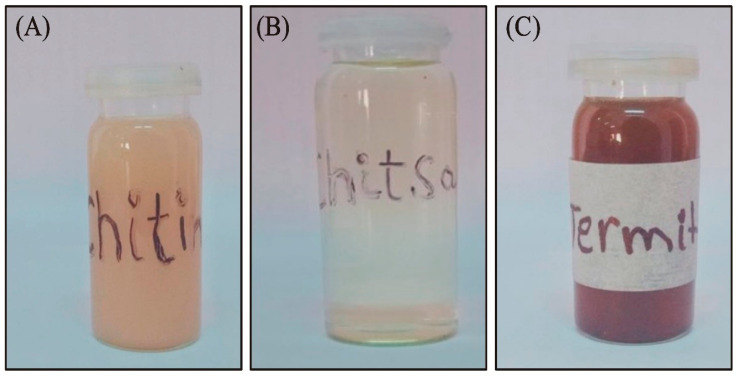
(**A**) Chitin solution (**B**) chitosan solution (**C**) termite’s chitin solution derived from *Periplaneta americana* and termites.

Approximately 420 *Periplaneta americana* and 220 termites were collected, and the specimens were allowed to air dry at room temperature in the entomology lab for about two weeks. All specimens were crushed using a sterilized mortar and weighed with a digital balance in the molecular genetics lab of Abdul Wali Khan University Mardan, KPK. A total of 10 g (5 g chitin, 5 g chitosan) of fine powder was obtained from termite and Periplaneta americana integument. Chitin was subjected to 500 mL of (2.07 mol/L) HCL for 30 min at 100 degrees Celsius to achieve demineralization. The pH was subsequently adjusted to 7, i.e., neutral pH, by soaking with distilled water. The dry powder of demineralized ingredients was subjected to 500 mL of (1 mol/L) NaOH for deproteinization. The chitin was subjected to 250 mL (0.6 mol/L) NaOH for 4 h at 100 degrees Celsius for deacetylation to obtain chitosan. After the alkali was carefully cleansed and drained using DW until the pH was less than 7.5, the samples were allowed to dry at room temperature [16] (Figure 1).

### 2.3. Physical Characterization

#### 2.3.1. FTI-R Analysis

The samples were examined by FT-IT spectroscopy at Bacha Khan University’s Department of Chemistry using a PerkinElmer spectrometer FT-IR SPECTRUM ONE (Spectra Lab Scientific Inc., Markham, ON, Canada) with a resolution of 4 cm^−1^ in the 4000 cm^−1^–0 cm^−1^ range. A 37 °C vacuum dryer setting was used to thoroughly dry the chitin and chitosan. The spectrum spans 4 cm^−1^ through 4000 cm^−1^. We identified certain functional groups within the samples via FT-IR spectroscopy on *Periplaneta americana*, chitin, and chitosan termite chitin [17].

#### 2.3.2. X-ray Powder Diffraction (XRD) Analysis

A JEOL JDX 3532 XRD was utilized to examine samples at the University of Peshawar (JEOL Ltd., Yokosuka, Japan). The crystallinity of distinct chitins and chitosan has been identified by the XRD method. The crystalline index (CrI) values of chitin and chitosan derived from *Periplaneta americana* and termites have been evaluated using the following formula [18].

$$Crl110=\frac{\left(I_{\left(110\right)}-I_{\left(am\right)}\right)}{I_{\left(110\right)}\;}\times 100$$

### 2.4. Biological Activities

#### 2.4.1. Assays for Analgesic Activity

A study was conducted using mice to evaluate the potential analgesic effects of chitin and chitosan extracts. For this, mice were divided into six groups. 1 mL of a 1% *v*/*v* normal saline solution was given to the 1st group, considered a control group. The 2nd group was treated with paracetamol and considered as a standard group. The remaining groups, 3, 4, 5, and 6, were treated with test samples at different concentrations (50 µL/mL, 100 µL/mL, and 500 µL/mL). Writhing was observed for five minutes after the acetic acid solution was injected [19]. The following formula was used to calculate the percentage of pain-relieving action.

$$Inhibition\;(\%)\;=\;\frac{Number\;of\;writhing\;in\;test\;group}{Number\;of\;writhing\;in\;the\;control\;group}\times 100$$

#### 2.4.2. In Vivo Assay for Anti-Inflammatory Effect

Mice were used to examine the extract’s anti-inflammatory potential as test model animals. Through an official agreement between the university and the Veterinary Research Institute (VRI) in Peshawar, twenty-five male Swiss albino mice weighing between 20 and 30 g were brought. The mice at Abdul Wali Khan University Mardan (AWKUM) were kept in polypropylene cages to give them time to adjust to their new environment. Temperature ranges of 24–26 °C, 40–45% humidity, and a 12-h light/dark cycle were maintained. The mice were starved for almost a night to ensure adequate anti-inflammatory effects. The AWKUM Zoology Department’s ethics committee approved the whole experiment to be conducted. Subsequently, xylene can cause inflammation [20].

To cause edema, a single drop of xylene was put on each side of the mice’s ears using a micropipette. The positive control group did not have any infections. The thickness was measured before using xylene as a medication. Fifteen minutes later, the ear turned red, a sign of irritation and the development of ear edema. Twelve mice were selected, and different treatment concentrations were given to four groups of three mice each. The right ear was treated with varying volumes of extract (50 µL/mL, 100 µL/mL, and 500 µL/mL) when it turned red; edema was observed after 15 min. None of the mice (negative control group) were given any treatment for their left ear. The measurement of the ear’s thickness was done by using a micro screw gauge at 1, 4, 12, and 24 h. Diclofenac sodium (standard drug) was given to each mouse’s right ear of the standard group. The control group was not assigned xylene, while the positive control remained untreated. Different extract concentrations (50 µL/mL, 100 µL/mL, and 500 µL/mL) of chitin and chitosan of *Periplaneta americana* and termites were given to the mice’s right ear. Micro screw gauges were used to measure ear edema after 1, 4, 12, and 24 h. A day later, the mice were given a small dose of anesthesia, and the ears were removed and preserved in 10% formalin. The mice’s ears were transported hygienically to the Ali Pathology Lab and Diagnostic Center in Islamabad, Pakistan, for histopathology examination. The following formula was used to calculate the percentages of edema inhibition.

$$\%\;Inhibition\;Ear\;edema=\frac{\left(Thickness\;of\;Control-Thickness\;of\;the\;treated\right)}{Thickness\;of\;the\;control\;group}\times 100$$

#### 2.4.3. Antipyretic Activity

The study evaluated the antipyretic potential of brewer’s-yeast-induced pyrexia in albino mice to assess its effectiveness in living organisms. To produce pyrexia in mice, a subcutaneous injection of 20% aqueous brewer’s yeast solution at a dose of 10 mL/kg was given. Each mouse’s temperature was measured using a lubricated rectal thermometer before applying the yeast solution. After eighteen hours, the mice’s body temperatures were again measured, and the mice’s body temperatures were recorded for four hours [21].

### 2.5. Statistical Analysis

One-way (ANOVA) analysis (n = 6) and SPSS ver 16.0 software (Chicago, IL, USA), Origin 7.5, and Graphad prism software 10.2.0 were used to analyze the data statistically.

## 3. Results and Discussion

### 3.1. Fourier Transforms Infrared Spectroscopy Analysis

FT-IR spectroscopy is a rapid and efficient technique for detecting functional groups within chitin and chitosan of *Periplaneta americana* and termite chitin. Using FT-IR spectra, two distinct groups (β and α) were differentiated, particularly concerning the division of the amide I band. The alpha form showed a split amide I band among 1650 and 1620 cm^−1^, even as the beta form revealed the closest amide I band at 1656 cm^−1^. Alpha chitin was detected commonly in arthropods. This was supported by bands at 1633.4 inside the FT-IR spectra of *Periplaneta americana*. Furthermore, extra bands were determined at various wavelengths: 1535 cm^−1^ (C=C, halogen groups), 3300 cm^−1^ (asymmetric N–H bond, carboxylic acid), 1411 cm^−1^ (twisting of CH_2_ and CH_3_) 2846 cm^−1^ (symmetric C–H), 1181 cm^−1^ (C–O–C bond), 2938 cm^−1^ (C–H bond), 1633 cm^−1^ (Diketone, N–H bond), 950 cm^−1^ (twisting of CH_3_ and O–H stretching), 1027 cm^−1^ (alkyl amine), and 1153 cm^−1^ (ester carbonyl institution C–O–C uneven bond). In the Fourier transforms infrared spectroscopy evaluation of chitosan, various bands were recognized: at 3304 cm^−1^ (N-H bond and carboxylic acid), 2846 cm^−1^ (related to symmetric C–H stretching), 1605 cm^−1^ (linked to diketones and N–H bonds), 2938 cm^−1^ (indicative of the C–H bond), 1467 cm^−1^ (representing CH_2_ and CH_3_, in particular, Nitrosamine), 1535 cm^−1^ (related to C=C and halogen groups), 1235 cm^−1^ (indicative of alkyl ketones and C–O–C uneven stretching), 720 cm^−1^ (indicative of a chloro compound), and 588 cm^−1^ (associated with C=C) 950 cm^−1^ (related to CH_3_ wagging and O–H). The presence of the amino group was located within the band at 1605 cm^−1^, confirming the fulfillment of the deacetylation method. Furthermore, functional bands at 3304, 2846 (C–H bond), and 1089 cm^−1^ (C–O bond) were also identified in the chitosan.

### 3.2. Termite Chitin FT-IR

**Figure 2:**
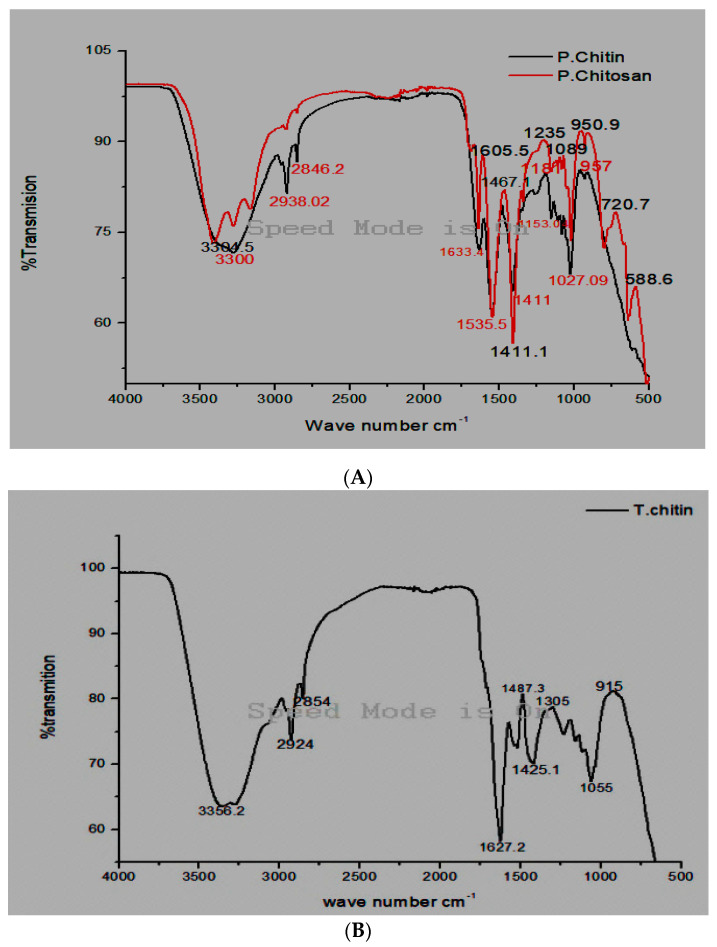
FT–IR spectra of (**A**) chitin and chitosan (**B**) termite chitin derived from *Periplaneta americana* and termites.

The Fourier transforms infrared spectroscopy spectrum of the chitin bands from termites revealed particular bands at numerous wavelengths, indicative of unique chemical bonds, 2924 cm^−1^ (C–H stretching), 3356 cm^−1^ (carboxyl acid), 2854 cm^−1^ (symmetric C–H stretching), 1425.1 cm^−1^ (C–O/C–H), 1627 cm^−1^ (Di ketone, N–H bond), 1487 cm^−1^ (alkyl ketone, CH_2_ ending and CH_3_ deformation, Nitrosamine), 1305 cm^−1^ (C–N stretching), 1055 cm^−1^ (polysaccharides), and 915 cm^−1^ (CH_3_ wagging, O–H stretching) [18]. Any other research through [22] concerned FT-IR analysis of house cricket chitin, confirming at various wavelengths: 3300, 2938, 2846.2, 1633. Bands at 1535.5, 1411, 1153.0, 1181, 1027, and 957 cm^−1^, which constitute O–H stretching, CH_2_ ending and CH_3_ deformation, halogen CH_2_ ending and CH_3_ deformation, N–H stretching, amides I, II, and III, alkyl ketone, Nitrosamine, C=C and C–O–C stretching [22] (Figure 2).

### 3.3. X-rays Diffraction (XRD) Analysis

**Figure 3:**
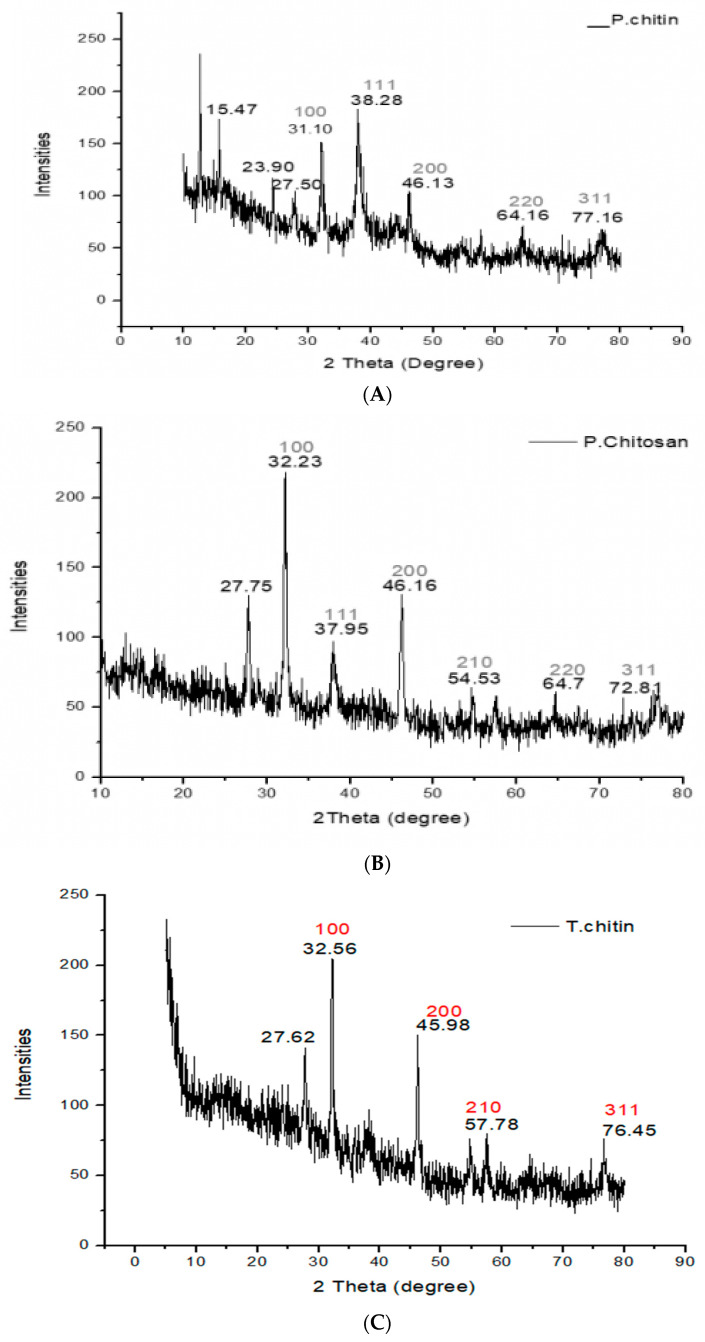
XRD analysis of (**A**) chitin, (**B**) chitosan, and (**C**) termite chitin derived from *Periplaneta americana* and termites.

The molecular and atomic arrangement of the crystals was determined using XRD analysis. For chitin, this was (31.10, 38.28, 46.13, 64.16, 77.16) for chitosan, it was (32.23, 37.95, 46.16, 54.53, 64.7, 72.81), and for termites, it was (32.56, 45.98, 57.78, 76.45). The crystals scattered light in different directions. X-ray diffraction analysis was used to investigate the crystal structure of the chitin and chitosan obtained from termites and *Periplaneta americana*. At 2Ø, the surface averaged cubic ray diffraction peaks of chitin and chitosan were discovered, and the corresponding surfaces at (100), (111), (200), (210), (220), and (311) were identified in it. Every peak and the JCPDS polymer standard file revealed consistency. These peaks demonstrated the crystalline chitin and chitosan in *Periplaneta americana* and termites (Figure 3).

### 3.4. Analgesic Activity

**Figure 4:**
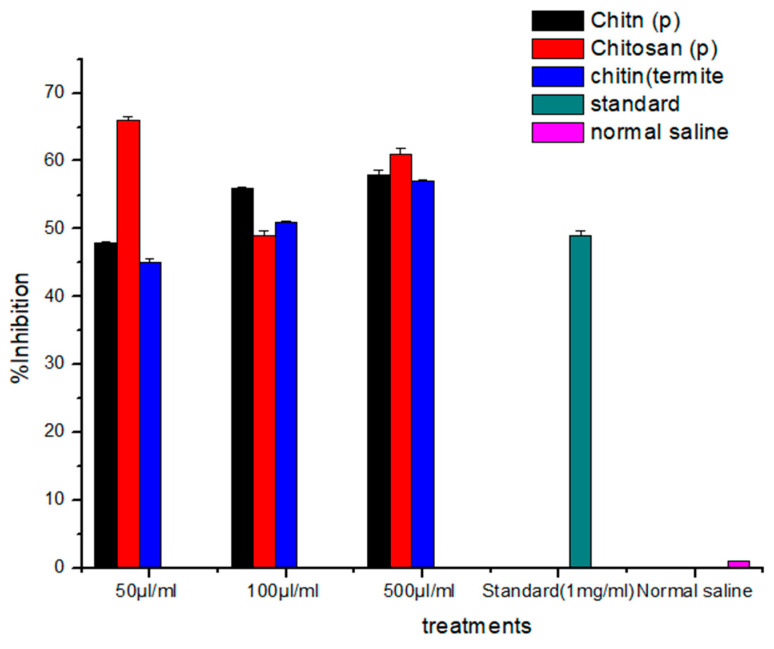
Analgesic activity graph of chitin and chitosan and termite’s chitin derived from *Periplaneta americana* and termites.

The analgesic potential of chitin and chitosan derived from termites and *Periplaneta americana* was evaluated to determine the pain-relieving properties. Four replicates of samples at different concentrations (50 µL/mL, 100 µL/mL, and 500 µL/mL) were taken. Chitin, chitosan, and termite chitin demonstrated the ability to decrease pain in mice. At a concentration of 50 µL/mL, chitin showed the most significant (*p* < 0.0001) inhibition, resulting in a decrease in writhing from an initial value of 69 to a final value of 41, approximately 46.06% inhibition. Moreover, the highest level of inhibition with chitosan occurred at a concentration of 100 µL/mL, resulting in a decrease in writhing from the initial value of 66 to a final value of 41, with a percent inhibition of approximately 46.06%. Termite chitin showed its maximum percent inhibition at 500 µL/mL and decreased from the initial value of 63 to 38 final values, with its maximum percent inhibition of about 42.69%. Termite chitin was found to have the highest percent inhibition (42.69%) among these comparative studies: chitin, chitosan, and termite chitin. Paracetamol, a common drug, showed a significant inhibition of 44.94%. The chitosan absorbed the H^+^ ions released in the inflamed areas [23]. Chitin was much more effective than chitosan in terms of analgesic effect. Compared to chitosan, chitin significantly reduced the number of writhings. Chitin absorbs bradykinin, so it works well (Table 1 and Figure 4).

Aranaz et al. [24] investigated the potential of chitin and chitosan and their analgesic properties. Their results showed that both chitin and chitosan had analgesic properties. They examined the effect of these biopolymers on inflammatory pain by the intramuscular injection of acetic acid. Their study showed that chitosan had a more substantial impact than chitin, probably due to these two polymers’ different mechanisms of action. The analgesic effect of chitosan was related to the production of proton ions at the site of inflammation. The control group (Group 1) showed curves after (2740.10)/5 min. Group 2 showed a 40.43% reduction in bending movements, which decreased to 140.21/5 min. The highest number of deviations was observed in mice in group 5 at a concentration of 300 mg/kg (210.13/5 min with a 45% reduction in inhibition. Mice in group 4 with a concentration of 200 mg/kg (280.32/5 min) followed closely with a 52.44% reduction in inhibition. Research showed that chitin has significantly stronger analgesic properties than chitosan. The increase in the effectiveness of chitin depends on the absorption of bradykinin, as a result of which the analgesic effect increases.

### 3.5. Anti-Inflammatory In Vivo Assay

**Figure 5:**
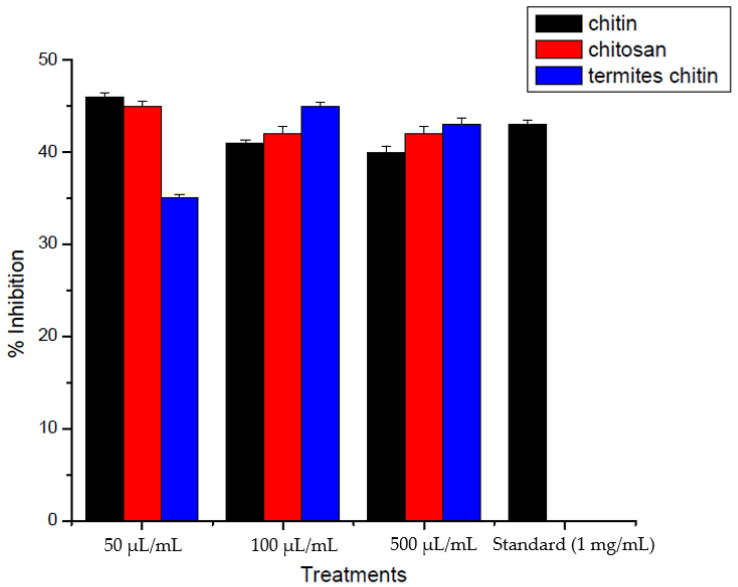
Anti-inflammatory graph of chitin, chitosan, and termite’s chitin extracted from *Periplaneta americana* and termites.

**Figure 6:**
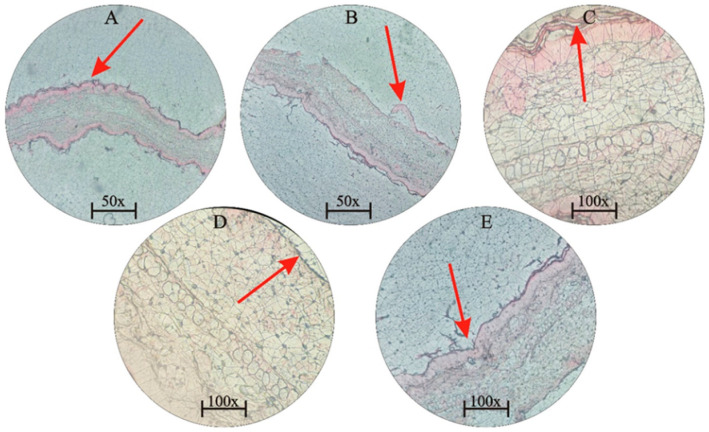
(**A**) Histological section of the ear at 50 µL/mL extracts (magnification power = 50×). (**B**) Extract inhibition at 100 µL/mL (magnification power = 50×). (**C**) Extract inhibition at 500 µL/mL (magnification power = 100×). (**D**) Termite’s chitin at 500 µL/mL (magnification power = 100×). (**E**) Shows a positive control (magnification power = 100×), which is an enlarged section of the tissue shown in (**B**) and demonstrates a parallel reduction in swelling in a different part of the same tissue. The red arrows indicate the reduction of swelling in specific areas.

One of the substances that caused inflammation was xylene [20]. The sides of both ears were treated with two drops of xylene. After 15 to 20 min, the ear became red due to inflamed vessels, and it was found that the mucus was thicker as a result of the xylene treatment. While the extract with different concentrations (50 μL/mL, 100 μL/mL, and 500 μL/mL), the standard group was treated with diclofenac sodium, a standard drug. The control group was not given xylene, while the positive control remained untreated. Different results were obtained at different concentrations. The mice showed a 28% reduction in thickness after 1 h at a concentration of 50 μL/mL. After 4 h, it showed a 34% reduction in thickness at 100 μL/mL. After 12 h, a 40% reduction occurred in the thickness, while after 24 h, at 500 μL/mL, there was a 46% reduction in thickness. The standard drug showed a 41% reduction in thickness. The greatest reduction in thickness was occurred with chitin at 500 μL/mL, followed by termite’s chitin at 500 μL/mL which showed a 44% reduction in thickness. In a comparative study, chitin showed the highest inhibition percentage (46.16%). Diclofenac sodium, a common drug, showed a percent inhibition of 41.94% (Figure 5 and Figure 6).

Tavaria et al. [25] worked on mice using. After administering croton oil, HMW chitosan showed a slight edema inhibition (22%) in comparison to the control group; however, this difference was not found to be significant (*p* > 0.05). Because it was an allergic reaction, croton oil was known to cause edema, while dexamethasone was a steroid that reduced inflammation. The primary irritating compounds in cotton oil are 12-o-tetracanoilphorbol-13-acetate (TPA) and other phorbol esters. Platelet activation factor and arachidonic acid were released when TPA stimulates protein kinase C, activating other enzymatic cascades such as mitogen-activated protein kinases and phospholipase A2. This series of events brought on vascular permeability, vasodilation, polymorphonuclear leukocyte movement, histamine release, and serotonin release.

Jin et al. [26] revealed that CCG might be a valuable candidate for an anti-allergic medication. Compound 48/80 caused systemic anaphylactic shock, and CCG decreased ear swelling reactions. Oral dosing or the topical application of CCG reduced IgE-mediated PCA. CCG therapy reduced the release of histamine and β-hexosaminidase from mast cells. By inhibiting NF-κB activation and IκBα phosphorylation, CCG also suppressed interleukin-1β production and mRNA expression caused by phorbol 12-myristate 13-acetate and calcium ionophore A23187. Moreover, CCG inhibited caspase-1 activation.

### 3.6. Antipyretic Assay

The brewer’s yeast method was used in this experiment. The animals were given brewer’s yeast in milliliters per kilogram or 20% of their body weight. Then, mice were given a therapeutic dose of chitin, chitosan, and termite chitin with different concentrations (50 μL/mL, 100 μL/mL, and 500 μL/mL). The body temperature was measured once an hour for four hours using a direct contact thermometer. The body temperature of the mice at 500 μL/mL of termite chitin, chitosan, and chitin (*Periplaneta americana*) was regulated in the third hour (Table 2).

## 4. Conclusions

Termites and *Periplaneta americana* are characterized by the production of chitin and chitosan. The synthesis of chitin and chitosan requires different treatments with acids and alkalis. Chitosan was obtained by the deacetylation of chitin. XRD analysis was used to determine the crystallinity of these biopolymers. The results showed that chitin and chitosan are arranged in a dense cubic configuration. According to FTIR, different types of functional groups were identified in the chitin and chitosan of Termites and *Periplaneta americana.* As a result of FTIR, various compounds such as ether alkanes, alkynes, alcohols, amides, carbonyls, alkyl ketones, halogens, and nitrosamines are found in the chitin and chitosan of termites and *Periplaneta americana*. These compounds hold potential for medical applications, aiding in developing safer medications and more efficient vaccines. Chitosan’s ability to absorb proton ions at sites of inflammation was observed to reduce acetic acid-induced writhing activity, demonstrating the substantial potential of chitin and chitosan in decreasing pain. Biological-based biopolymers, chitin, and chitosan possess anti-inflammatory and antipyretic properties that could offer various benefits. Chitin exhibits the highest degree of analgesic activity compared to chitosan. Both chitin and chitosan demonstrate anti-inflammatory effects, with chitosan absorbing proton ions at sites of inflammation, while chitin effectively inhibits ear edema and elicits an analgesic response in mice. This study revealed antipyretic activity, with termite chitin demonstrating the most significant effect at a concentration of 500 µL/mL, followed by chitosan and chitin at 100 µL/mL. Chitin and chitosan are derived from termites and *Periplaneta americana* as natural anti-inflammatory compounds, implying their prospective uses in anti-inflammatory, antipyretic, and analgesic capabilities.

## Figures and Tables

**Table 1:** Statistical analysis related to analgesic activity.

	Groups	Treatment	Dose mL or mg/kg	Initial Value	Final Value	% Analgesia
Analgesic Chitin (chi)	Gr.1	chi1	50 μL/mL	68.25 ± 0.95	42.75 ± 1.70	48.02%
	Gr.2	chi2	100 μL/mL	75.00 ± 2.94	50.00 ± 2.58	56.17%
	Gr.3	chi3	500 μL/mL	76.00 ± 5.35	52.25 ± 2.75	58.70%
Analgesic chitosan (chs)	Gr.1	chs1	50 μL/mL	84.25 ± 3.94	59.25 ± 1.70	66.56%
	Gr.2	chs2	100 μL/mL	66.50 ± 3.31	44.25 ± 2.75	49.71%
	Gr.3	chs3	500 μL/mL	74.25 ± 2.75	55.00 ± 3.65	61.79%
Analgesic termite’s chitin (ter chi)	Gr.1	ter chi1	50 μL/mL	62.50 ± 3.00	40.50 ± 1.29	45.50%
	Gr.2	ter chi2	100 μL/mL	63.00 ± 3.16	45.50 ± 3.41	51.11%
	Gr.3	ter chi3	500 μL/mL	72.25 ± 6.50	51.00 ± 8.83	57.30%
	Gr.4	Standard	1 mL	54.50 ± 4.14	44.25 ± 4.34	49.71%
	Gr.5	Control	1% *v*/*v*	88.75 ± 0.50	87.25 ± 0.95	73.68%

**Table 2:** Antipyretic potential using Pariplaneta americana chitin, chitosan, and termite’s chitin samples.

Drug	Dose (μL/mL or mg/mL)	Temperature (°F) before Applying Yeast	Temperature (°F) after Using the Sample				
			0 h	1 h	2 h	3 h	4 h
Negative Control (Normal Saline)	10% *v*/*v*	98.7	101	101	101	101	101
Positive Control (Paracetamol)	1 mg/mL	98.7	100	98.8	98.7	98.7	98.8
Chitin	50 µL/mL, 100 µL/mL and 500 µL/mL	98.7	100, 99.5, 98.6	99.7, 99.5, 98.2	100, 99, 98.5	100, 98, 99.3	100.2, 99.1, 98.6
Chitosan	50 µL/mL, 100 µL/mL and 500 µL/mL	98.7	100.5, 99, 100	100.1, 99.5, 99	100.1, 99.4, 98	100.6, 98, 99.1	100.3, 99, 98
termite’s chitin	50 µL/mL, 100 µL/mL and 500 µL/mL	98.7	100.5, 99.9, 99.5	100, 99.6, 99.1	100, 99.1, 99	100.5, 99.9, 98	100, 99.2, 99.1

## Data Availability

All data generated or analyzed during this study are included in this published article.

## References

[1] Croisier F., Jérôme C. (2013). Chitosan-based biomaterials for tissue engineering. Eur. Polym. J..

[2] Kumari S., Rath P., Kumar A.S.H., Tiwari T.N. (2015). Extraction and characterization of chitin and chitosan from fishery waste by chemical method. Environ. Technol. Innov..

[3] Berger L.R.R., Stamford T.C.M., de Oliveira K.Á.R., Pessoa A.D.M.P., de Lima M.A.B., Pintado M.M.E., Câmara M.P.S., de Oliveira Franco L., Magnani M., de Souza E.L. (2018). Chitosan produced from Mucorales fungi using agroindustrial by-products and its efficacy to inhibit Colletotrichum species. Int. J. Biol. Macromol..

[4] Periayah M.H., Halim A.S., Saad A.Z.M. (2016). Chitosan: A promising marine polysaccharide for biomedical research. Pharmacogn. Rev..

[5] Goy R.C., Britto D.D., Assis O.B. (2009). Assis, A review of the antimicrobial activity of chitosan. Polímeros.

[6] Huang R., Liu Q., Huo J., Yang B. (2017). Green preparation of a cellulose nanocrystals/polyvinyl alcohol composite superhydrophobic coating. RSC Adv..

[7] Muzzarelli R.A., Boudrant J., Meyer D., Manno N., DeMarchis M., Paoletti M.G. (2012). Current views on fungal chitin/chitosan, human chitinases, food preservation, glucans, pectins and inulin: A tribute to Henri Braconnot, precursor of the carbohydrate polymers science, on the chitin bicentennial. Carbohydr. Polym..

[8] Vinsova J., Vavrikova E. (2011). Chitosan derivatives with antimicrobial, antitumour and antioxidant activities—A review. Curr. Pharm. Des..

[9] Mohammed M.H., Williams P.A., Tverezovskaya O. (2013). Extraction of chitin from prawn shells and conversion to low molecular mass chitosan. Food Hydrocoll..

[10] Mourya V.K., Inamdar N.N. (2008). Chitosan-modifications and applications: Opportunities galore. React. Funct. Polym..

[11] Philippova O.E., Korchagina E.V., Volkov E.V., Smirnov V.A., Khokhlov A.R., Rinaudo M. (2012). Aggregation of some water-soluble derivatives of chitin in aqueous solutions: Role of the degree of acetylation and effect of hydrogen bond breaker. Carbohydr. Polym..

[12] Roy J.C., Salaün F., Giraud S., Ferri A., Chen G., Guan J. (2017). Solubility of chitin: Solvents, solution behaviors, and their related mechanisms. Solubility Polysacch..

[13] Mullins D.E. (2015). Physiology of environmental adaptations and resource acquisition in cockroaches. Annu. Rev. Entomol..

[14] Wanule D., Balkhande J.V., Ratnakar P.U., Kulkarni A.N., Bhowate C.S. (2014). Extraction, and FTIR analysis of chitosan from American cockroach, *Periplaneta americana*. Extraction.

[15] Bignell D.E. (2016). The role of symbionts in the evolution of termites and their rise to ecological dominance in the tropics. The Mechanistic Benefits of Microbial Symbionts.

[16] Mohan K., Ganesan A.R., Muralisankar T., Jayakumar R., Sathishkumar P., Uthayakumar V., Chandirasekar R., Revathi N. (2020). Recent insights into the extraction, characterization, and bioactivities of chitin and chitosan from insects. Trends Food Sci. Technol..

[17] Acay H., Baran M.F. (2019). Investigating antimicrobial activity of silver nanoparticles produced through the green synthesis using leaf extract of common grape (*Vitis vinifera*). Appl. Ecol. Environ. Res..

[18] Kaya M., Baran T., Asan-Ozusaglam M., Cakmak Y.S., Tozak K.O., Mol A., Mentes A., Sezen G. (2015). Extraction and characterization of chitin and chitosan with antimicrobial and antioxidant activities from cosmopolitan *Orthoptera* species (Insecta). Biotechnol. Bioprocess Eng..

[19] Khan M.S., Ullah S. (2018). Analgesic, anti-inflammatory, antioxidant activity and phytochemical screening of *Dryopteris blanfordii* plant. J. Pharmacogn. Phytochem..

[20] Oh Y.C., Jeong Y.H., Cho W.K., Ha J.H., Gu M.J., Ma J.Y. (2015). Anti-inflammatory and analgesic effects of pyeongwisan on LPS-stimulated murine macrophages and mouse models of acetic acid-induced writhing response and xylene-induced ear edema. Int. J. Mol. Sci..

[21] Sulaiman S., Ahmad S., Naz S.S., Qaisar S., Muhammad S., Ullah R., Al-Sadoon M.K., Gulnaz A. (2022). Synthesis of zinc oxide-based etoricoxib and montelukast nanoformulations and their evaluation through analgesic, anti-inflammatory, antipyretic, and acute toxicity activities. J. King Saud. Univ. Sci..

[22] Ibitoye E.B., Lokman I.H., Hezmee M.N.M., Goh Y.M., Zuki A.B.Z., Jimoh A.A. (2018). Extraction and physicochemical characterization of chitin and chitosan isolated from house cricket. Biomed. Mater..

[23] Şenel S., McClure S.J. (2004). Potential applications of chitosan in veterinary medicine. Adv. Drug Deliv. Rev..

[24] Aranaz I., Mengíbar M., Harris R., Paños I., Miralles B., Acosta N., Galed G., Heras Á. (2009). Functional characterization of chitin and chitosan. Curr. Chem. Biol..

[25] Tavaria F., Jorge M.P., Ruiz L.T., Pintado M.E., Carvalho J.E. (2016). Anti-proliferative, anti-inflammatory, anti-ulcerogenic and wound healing properties of chitosan. Curr. Bioact. Compd..

[26] Jin S.E., Jung J., Jun J., Jeon D.W., Kim H.M., Jeong H.J. (2012). Anti-allergic activity of crystallinity controlled N-acetyl glucosamine. Immunopharmacol. Immunotoxicol..

