# Correction: Asad et al. Anti-Inflammatory, Antipyretic, and Analgesic Potential of Chitin and Chitosan Derived from Cockroaches (*Periplaneta americana*) and Termites. *J. Funct. Biomater.* 2024, *15*, 80

**DOI:** 10.3390/jfb15090255

**Published:** 2024-09-02

**Authors:** Khushbakht Asad, Sumaira Shams, Eliana Ibáñez-Arancibia, Patricio R. De los Ríos-Escalante, Farhad Badshah, Farooq Ahmad, Muhammad Salman Khan, Asar Khan

**Affiliations:** 1 Department of Zoology, Abdul Wali Khan University Mardan, Mardan 23200, Pakistanasar1056@gmail.com (A.K.); 2 PhD Program in Sciences Mentioning Applied Molecular and Cell Biology, La Frontera University, Temuco 4780000, Chile; 3 Laboratory of Engineering, Biotechnology and Applied Biochemistry—LIBBA, Department of Chemical Engineering, Faculty of Engineering and Science, La Frontera University, Temuco 4780000, Chile; 4 Department of Biological and Chemical Sciences, Faculty of Natural Resources, Catholic University of Temuco, Temuco 4780000, Chile; prios@uct.cl; 5 Nucleus of Environmental Sciences, Faculty of Natural Resources, Catholic University of Temuco, Temuco 4780000, Chile; 6 State Key Laboratory of Animal Biotech Breeding, Institute of Animal Science, Chinese Academy of Agricultural Sciences, Beijing 100193, China

In the original publication [1], there was a mistake in Figure 6B as published. The original caption did not fully explain the content and significance of the images. The dual arrows were not clearly explained. The images did not include length scales. There was a discrepancy in the magnification levels between images A/B and C/D, which hindered proper comparison. Higher magnification images were difficult to interpret, with one arrow incorrectly pointing out of the field of view and the unclear characteristics of swollen versus non-swollen tissue. There was no positive control included to clearly demonstrate what swollen tissue should look like, especially in images C/D. The corrected figure and legend appear below.

The authors state that the scientific conclusions are unaffected. This correction was approved by the Academic Editor. The original publication has also been updated.

## Figures and Tables

**Figure 6:**
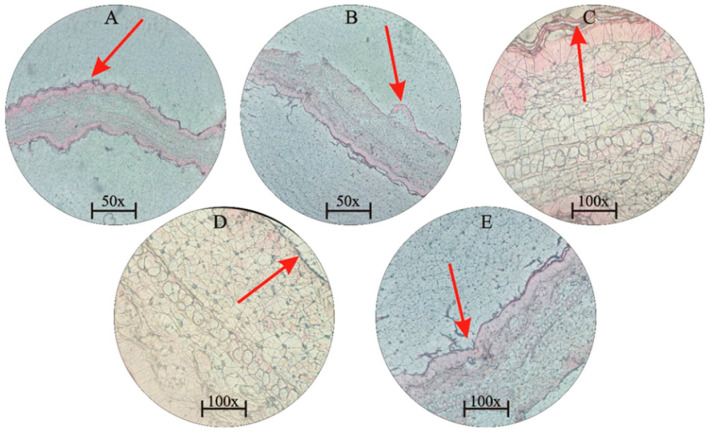
(**A**) Histological section of the ear at 50 µL/mL extracts (magnification power = 50×). (**B**) Extract inhibition at 100 µL/mL (magnification power = 50×). (**C**) Extract inhibition at 500 µL/mL (magnification power = 100×). (**D**) Termite’s chitin at 500 µL/mL (magnification power = 100×). (**E**) Shows a positive control (magnification power = 100×), which is an enlarged section of the tissue shown in (**B**) and demonstrates a parallel reduction in swelling in a different part of the same tissue. The red arrows indicate the reduction of swelling in specific areas.
